# Administration of single-dose GnRH agonist in the luteal phase in ICSI cycles: a meta-analysis

**DOI:** 10.1186/1477-7827-8-107

**Published:** 2010-09-08

**Authors:** João Batista A Oliveira, Ricardo Baruffi, Cláudia G Petersen, Ana L Mauri, Mario Cavagna, José G Franco

**Affiliations:** 1 Department of Gynecology and Obstetrics, Botucatu Medical School São Paulo State University - UNESP Sao Paulo, Brazil; 2 Center for Human Reproduction Prof. Franco Jr., Ribeirao Preto, Sao Paulo, Brazil; 3 Paulista Center for Diagnosis Research and Training, Ribeirao Preto, Sao Paulo, Brazil

## Abstract

**Background:**

The effects of gonadotrophin-releasing hormone agonist (GnRH-a) administered in the luteal phase remains controversial. This meta-analysis aimed to evaluate the effect of the administration of a single-dose of GnRH-a in the luteal phase on ICSI clinical outcomes.

**Methods:**

The research strategy included the online search of databases. Only randomized studies were included. The outcomes analyzed were implantation rate, clinical pregnancy rate (CPR) per transfer and ongoing pregnancy rate. The fixed effects model was used for odds ratio. In all trials, a single dose of GnRH-a was administered at day 5/6 after ICSI procedures.

**Results:**

All cycles presented statistically significantly higher rates of implantation (P < 0.0001), CPR per transfer (P = 0.006) and ongoing pregnancy (P = 0.02) in the group that received luteal-phase GnRH-a administration than in the control group (without luteal-phase-GnRH-a administration). When meta-analysis was carried out only in trials that had used long GnRH-a ovarian stimulation protocol, CPR per transfer (P = 0.06) and ongoing pregnancy (P = 0.23) rates were not significantly different between the groups, but implantation rate was significant higher (P = 0.02) in the group that received luteal-phase-GnRH-a administration. On the other hand, the results from trials that had used GnRH antagonist multi-dose ovarian stimulation protocol showed statistically significantly higher implantation (P = 0.0002), CPR per transfer (P = 0.04) and ongoing pregnancy rate (P = 0.04) in the luteal-phase-GnRH-a administration group. The majority of the results presented heterogeneity.

**Conclusions:**

These findings demonstrate that the luteal-phase single-dose GnRH-a administration can increase implantation rate in all cycles and CPR per transfer and ongoing pregnancy rate in cycles with GnRH antagonist ovarian stimulation protocol. Nevertheless, by considering the heterogeneity between the trials, it seems premature to recommend the use of GnRH-a in the luteal phase. Additional randomized controlled trials are necessary before evidence-based recommendations can be provided.

## Background

The depletion of granular cells due to follicular aspiration and the suppression of the release of luteinizing hormone (LH) by analogues (agonists and antagonists) of gonadotrophin-releasing hormone (GnRH) have been associated with a defect of the luteal phase in cycles of assisted reproduction. Furthermore, controlled ovarian stimulation was shown to be capable of accelerating endometrial maturation, hindering receptivity to embryo implantation[1]. Thus, through the years, there has been a continuous concern about the occurrence of luteal phase deficiency, principally in IVF/ICSI cycles [2,3], making the utilization of hCG, progesterone and sometimes estradiol (E2) a routine procedure in assisted reproduction technology (ART) to support the luteal phase. Recently, attention has been given to therapy with gonadotrophin-releasing hormone agonist (GnRH-a).

The effects of GnRH-a administration in the luteal phase has been the focus of different studies. Lemay et al. [4,5] suggested that GnRH-a can act as a luteolytic agent due to desensitization of GnRH receptors. Furthermore, Dubourdieu et al.[6] and Herman et al.[7] reported deterioration of corpus luteum function with the administration of GnRH-a. However, attempts to interrupt pregnancy or even prevent implantation have not been impressive[8,9]. On the other hand, a series of studies show that the inadvertent administration of GnRH-a in the luteal phase does not compromise the continuity of pregnancy, and suggested, to the contrary, a possible favorable effect on implantation [10-14]. Recently, different studies analyzing single [15-18] or multiple administrations [19-22] of medication have, in fact, suggested a beneficial effect in supporting the luteal phase. The mechanism of the presumed beneficial effect is poorly defined. It was suggested that GnRH-a can collaborate in the maintenance of the corpus luteum, acting directly on the endometrium via local receptors, a direct effect on the embryos or by some combination of these possibilities. On the other hand, other authors did not confirm positive action from the administration of GnRH-a in the luteal phase [23-25].

This meta-analysis aimed to evaluate the effect of the administration of single-dose GnRH-a in the luteal phase on IVF/ICSI clinical outcomes in ovarian stimulation protocols using GnRH antagonist (GnRH-ant) multi low-dose ovarian stimulation protocol or long GnRH-a ovarian stimulation protocol.

## Methods

### Criteria for considering studies for this meta-analysis

All published and ongoing randomized controlled trials assessing the effect of single dose GnRH-a administration in luteal support on IVF/ICSI outcomes were included. Frozen embryo replacement and egg donation cycles were not included. Due to the large difference in GnRH-a application schemes, studies with multiple applications of GnRH-a in the luteal phase were excluded.

### Outcome measures

The outcome measures used for this meta-analysis were implantation rate, clinical pregnancy rate (CPR) per transfer and ongoing pregnancy rate.

### Identification of studies

Search strategies included online surveys of databases (MEDLINE, EMBASE, Science Citation Index, Cochrane Controlled Trials Register and Ovid) from 1980 to 2010. There was no language restriction. The following medical subject headings and text words were used: GnRH agonist, luteal phase, luteal phase support, luteal phase administration, IVF, ICSI and randomized controlled trial. The principal inclusion criterion was randomized controlled trial (RCT).

### Search results

**Figure 1:**
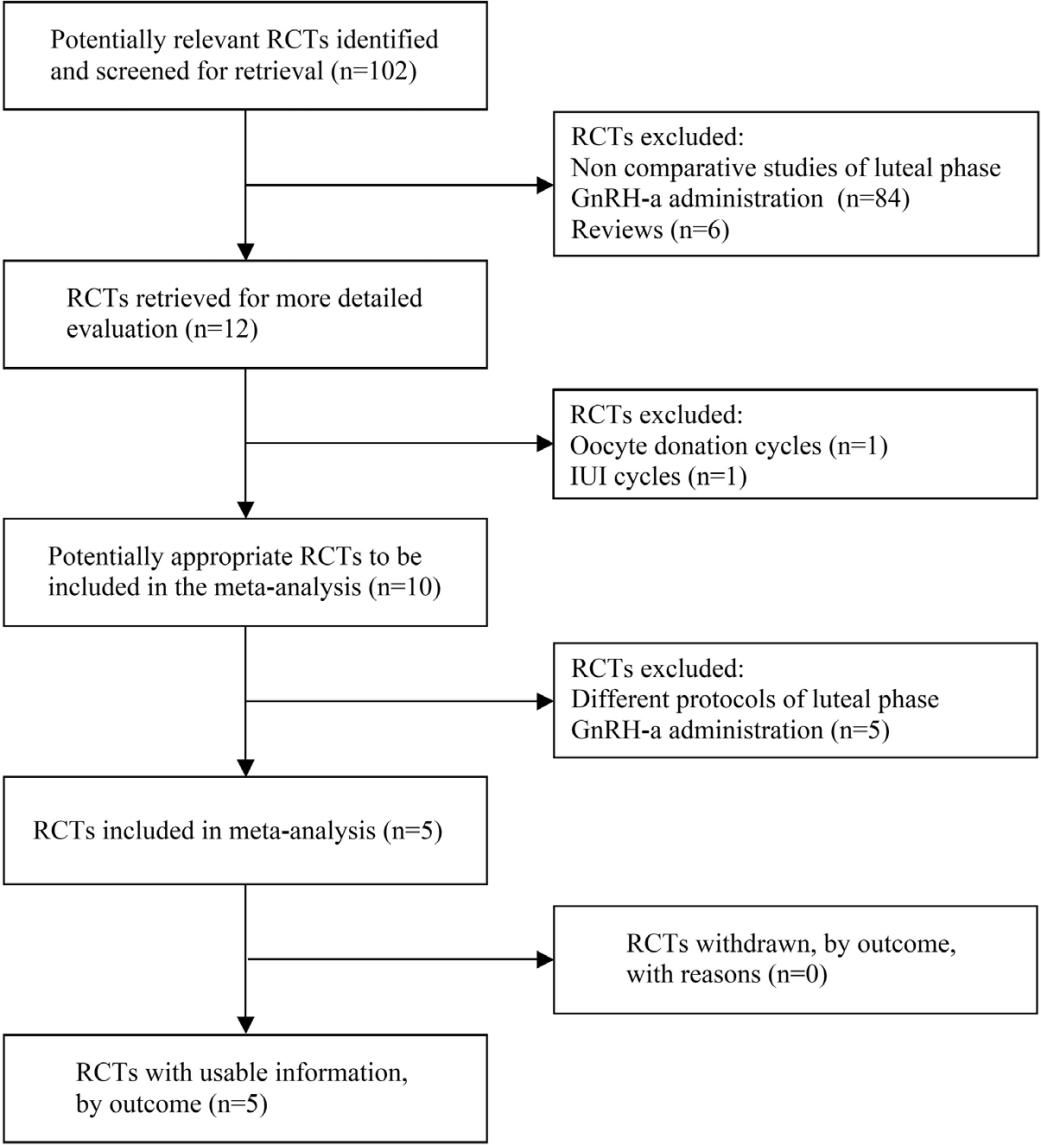
**QUOROM statement flow diagram illustrating selection of trials included in the meta-analysis**. RCT: randomized controlled trial.

Among the 12 potentially relevant studies retrieved, a total of 5 trials fulfilled the inclusion criteria [15,16,18,23,24]. A flow diagram of the selection process is shown in Fig 1.

### Description of the studies, validity assessment and data extraction

Each trial was assessed independently by three reviewers (JBAO, RB and JGF) and ranked for its methodological rigor and its potential to introduce bias. Missing data were obtained from the authors when possible.

In two trials [16,24] the long GnRH-a (leuprolide: 1, buserelin: 1) ovarian stimulation protocol starting in the mid-luteal phase of the preceding cycle was used as a reference treatment, in two [15,23] the GnRH-ant (cetrorelix: 1, cetrorelix/ganirelix: 1) multi low-dose ovarian stimulation protocol was applied and in one [18] both ovarian stimulation protocol types were used. In all studies ICSI was performed. Table 1 summarizes the main characteristics of the included trails.

**Table 1:** Main characteristics of randomized controlled trials (RCT) on the use of GnRH in luteal phase

Trial	Randomization	Protocol stimulation	Luteal Phase			Results
			Study group GnRH-a	Control group	Others medicines (all patients)	
Tesarik et al., 2006 [18]	Embryo transfer day Computer generated list Opaque envelopes Blinding	GnRH-a long protocol/GnRH-ant multiple dose + r-FSH/HMG + r-HCG	n:150 GnRH-a long protocol) n:150 GnRH-ant protocol Single injection Dose:0.1 mg/triptorelin Day 6 after ICSI	n:150 (GnRHa-long protocol) n:150 (Antagonist protocol) Placebo	E2 valerate (4 mg) + Vaginal micronized progesterone(400 mg) + r-HCG (single dose)	Improvement implantation/live birth rates
Ata et al., 2008 [24]	Embryo transfer day Computer generated list Opaque envelopes Blinding	GnRH-a long protocol + r-FSH + HCG	n:285 Single injection Dose:0.1 mg/triptorelin Day 6 after ICSI	n:285 Placebo	Vaginal progesterone gel/90 mg	No differences
Isik et al., 2009 [15]	Embryo transfer day Computer generated list Blinding	GnRH-ant multiple dose + r-FSH/HMG + hCG/r-hCG	n:74 Single injection Dose:0.5 mg/leuprolide Day 6 after ICSI	n:80 No placebo	Vaginal micronized progesterone(600 mg) + HCG(single dose)	Improvement implantation/clinical pregnancy rates
Razieh et al., 2009 [16]	Drawing piece of paper from a bag	GnRH-a long protocol + r-FSH + HCG	n:90 Single injection Dose:0.1 mg/triptorelin Day 5/6 after ICSI	n:90 Placebo	Vaginal micronized progesterone(800 mg)	Improvement implantation/clinical pregnancy rates
Ata and Urman, 2010 [23]	Embryo transfer day Computer generated list Opaque envelopes Blinding	GnRH-ant multiple dose + r-FSH/HMG + r-HCG	n:38 Single injection Dose:0.1 mg/triptorelin Day 6 after ICSI	n:52 Placebo	Vaginal progesterone gel/90 mg	Lower Implantation/ongoing pregnancy rates

### Description of the studies

Tesarik et al., 2006 [18] - This prospective randomized study evaluates the effect of GnRH-a administered in the luteal phase on ICSI outcomes in both GnRH-a- and GnRH-ant-treated ovarian stimulation protocols. Six hundred women (300 using a long GnRH-a protocol and 300 using a GnRH-ant protocol) were enrolled. Patients treated with each of these two protocols were randomly assigned to receive a single injection of GnRH-a (0.1 mg of triptorelin) or placebo 6 days after ICSI. Irrespective of whether GnRH-a was used as luteal-phase support or not, all women were given 4 mg of E2 valerate daily, 400 mg of vaginal micronized progesterone daily from the day of oocyte recovery for 17 days and an injection of 250 μg of HCG on the day of embryo transfer. In the results it was observed in GnRH-a-treated ovarian stimulation cycles a significant improvement of implantation and live birth in luteal-phase GnRH-a group as compared with placebo. In GnRH-ant-treated ovarian stimulation cycles, luteal-phase GnRH-a also increased ongoing pregnancy rate.

Ata et al., 2008 [24] - This double blind, randomized, placebo controlled trial evaluates whether a single dose GnRH-a administered 6 days after ICSI increases ongoing pregnancy rates in cycles stimulated with the long GnRH-a protocol. Five hundred and seventy women were included. In addition to routine luteal phase support with vaginal progesterone gel (90 mg), women were randomized to receive a single 0.1 mg dose of triptorelin or placebo 6 days after ICSI. Randomization was done on the day of ET according to a computer generated randomization table. There were 89 (31.2%) ongoing pregnancies in the GnRH agonist group, and 84 (29.5%) in the control group. Implantation, clinical pregnancy and multiple pregnancy rates were likewise similar in the GnRH agonist and placebo groups.

Isik et al., 2009 [15] - The study population consisted of 164 patients who underwent ICSI after ovulation induction by gonadotrophins and GnRH-ant for the prevention of a premature LH surge. For luteal-phase support, all the cases received intravaginal 600 mg micronized progesterone. In this prospective study, patients were randomly assigned to two groups. In one group, patients received an additional single dose of GnRH-a (0.5 mg leuprolide acetate) subcutaneously on day 6 after ICSI, whereas the patients in the other group did not. Although the number of embryos transferred and the grade of the embryos were similar in the two groups, the patients in the luteal-phase agonist group had significantly higher rates of implantation and clinical pregnancy rates (P < 0.05).

Razieh et al., 2009 [16] - The aim of this study was to assess the effect of single dose GnRH-a administered 3 days after embryo transfer, as luteal phase support, on ICSI cycles stimulated with the long GnRH-a protocol. One hundred and eighty women were enrolled. Patients were randomly assigned to receive a single dose of GnRH-a (0.1 mg of triptorelin) or placebo. The luteal phase was supported by administration of progesterone 800 mg daily in all the cases. It was observed that the patients in the luteal-phase GnRH-a group had a significant improvement in implantation rate (12.3% vs. 7.3%) and clinical pregnancy rate (25.5% vs. 10.0%) as compared with placebo.

Ata and Urman, 2010 [23] - This trial evaluated whether a single dose GnRH-a administered 6 days after ICSI increases ongoing pregnancy rates in cycles stimulated with the long GnRH-ant protocol. Ninety women were included. In addition to routine luteal phase support with vaginal progesterone gel (90 mg), women were randomized to receive a single 0.1 mg dose of triptorelin or placebo 6 days after ICSI. In the results it was observed a significant reduction in implantation and ongoing pregnancy rates in luteal GnRH-a group as compared with placebo. Clinical pregnancy rates were similar in the GnRH-a and placebo groups.

### Statistical analysis

Data management and analysis were conducted using the StatsDirect statistical software (Cheshire, UK). The fixed effect model was used for odds ratio (OR) and the effectiveness was evaluated by the Mantel-Haenszel method. A confidence interval (CI) was calculated using the variance formula of Robins, Breslow and Greenland. A chi-squared test statistic was used with its associated probability that the pooled OR was equal to 1. The measure of heterogeneity (non-combinability) was evaluated by Cochran's Q, the Breslow-Day and I^2 ^tests. A non-significant result (i.e. lack of heterogeneity) indicates that no trial had an OR significantly worse or better than the overall common OR obtained by pooling the data. Since a fixed effects model has been employed herein, it is important to acknowledge that inferences refer only to the particular studies included in the analysis. Meta-analysis used in this manner is simply a device to pool the information from the various studies to provide a composite finding, but only for those studies. Since many of the preceding analyses contained only two or three studies, it was decided to derive the inferences from a fixed-effects model.

## Results

### Implantation rate (Fig 2)

**Figure 2:**
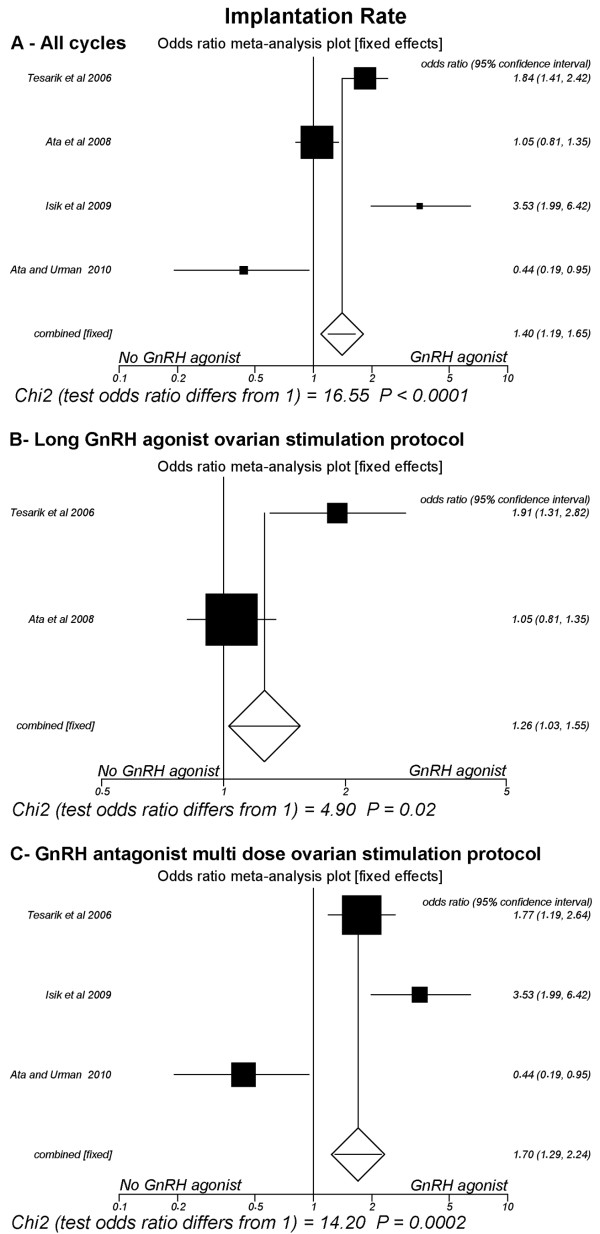
**Fixed-effect model**. Forest plot for implantation rates. A: All cycles; B: Only cycles with long GnRH agonist ovarian stimulation protocol; C: Only cycles with GnRH antagonist multi low-dose ovarian stimulation.

Four studies were included [15,18,23,24]. The pooled implantation rate was significantly higher in the group of patients that received GnRH-a in the luteal phase (24.4%, 411/1686) than in the group that did not receive this hormone agonist (18.6%, 335/1798) (P < 0.0001; OR = 1.40, 95% CI 1.19, 1.65). There was heterogeneity in this comparison (Breslow-Day = 31.52, df = 3, P < 0.0001; Cochran Q = 30.40, df = 3, P < 0.0001; I^2 ^= 90.1%, 95% CI = 75.6% to 94.5%).

In the subgroup of trials where the long GnRH-a ovarian stimulation protocol was used [18,24], the pooled implantation rate was significantly greater in the group of patients that received GnRH-a in the luteal phase (24%, 259/1080) than in the group that did not receive it (20%, 224/1122) (P = 0.02; OR = 1.26, 95% CI 1.03, 1.54). There was heterogeneity in this comparison (Breslow-Day = 7.25, df = 1, P = 0.007; Cochran Q = 7.21, df = 1, P = 0.007). Similarly, in the subgroup of trials where the GnRH-ant multi low-dose ovarian stimulation protocol was used [15,18,23], the pooled implantation rate was also significantly higher in the group of patients that received GnRH-a in the luteal phase (25.1%, 152/606) than in the group not administered this substance (16.4%, 111/676) (P = 0.0002; OR = 1.70, 95% CI 1.29, 2.23). There was heterogeneity in this comparison (Breslow-Day = 20.46, df = 2, P < 0.0001; Cochran Q = 20.17, df = 2, P <0.0001; I^2 ^= 90.1%, 95% CI = 66.9% to 95%)

### Clinical pregnancy rate per transfer (Fig 3)

**Figure 3:**
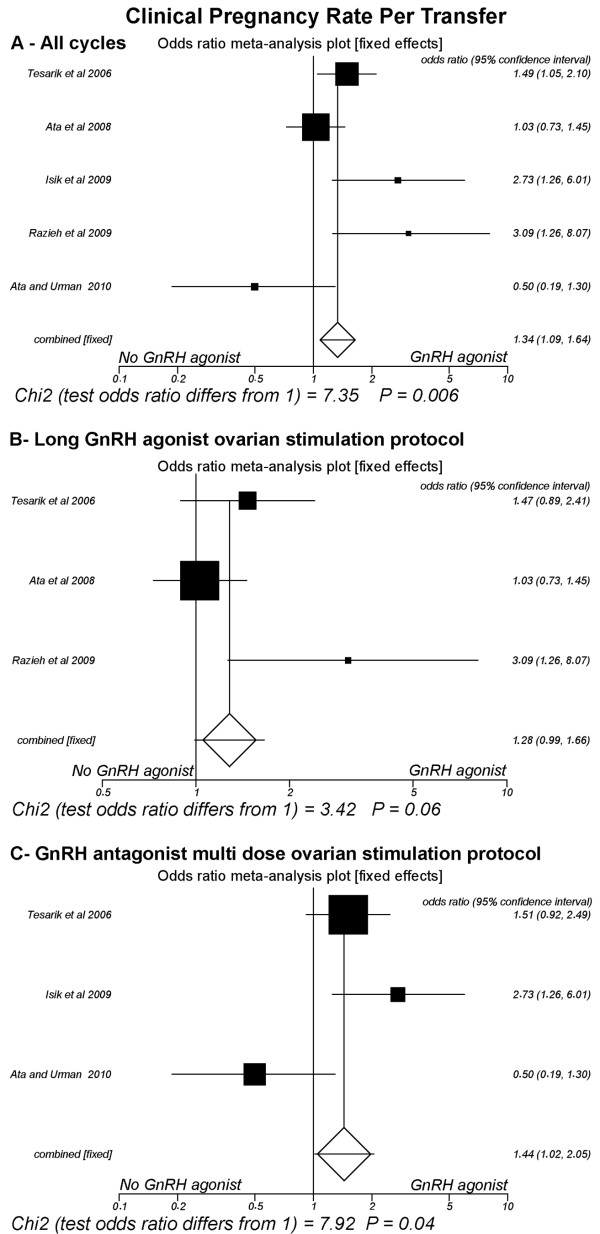
**Fixed-effect model**. Forest plot for clinical pregnancy rates per transfer. A: All cycles; B: Only cycles with long GnRH agonist ovarian stimulation protocol; C: Only cycles with GnRH antagonist multi low-dose ovarian stimulation.

Five studies were included [15,16,18,23,24]. The pooled CPR per transfer was significantly higher in the group of patients administered GnRH-a in the luteal phase (42.4%, 328/773) than in the group that did not receive it (35.7%, 283/793) (P = 0.006; OR = 1.33, 95% CI 1.08, 1.64). There was heterogeneity in this comparison (Breslow-Day = 15.65, df = 4, P = 0.003; Cochran Q = 15.32, df = 4, P = 0.004; I^2 ^= 73.9%, 95% CI = 0% to 87.5%).

However, in the subgroup of trials where the long GnRH-a ovarian stimulation protocol was used [16,18,24], the pooled CPR per transfer did not differ significantly between the group of patients that received GnRH-a in the luteal phase (42%, 217/516) versus the group that did not receive it (36.4%, 188/517) (P = 0.06; OR = 1.28, 95% CI 0.99, 1.65), a comparison with heterogeneity (Breslow-Day = 6.42, df = 2, P = 0.04; Cochran Q = 6.25, df = 2, P = 0.04; I^2 ^= 68%, 95% CI = 0% to 88.6%). On the other hand, in the subgroup of trials where the GnRH-ant ovarian stimulation protocol was used [15,18,23], the CPR per transfer was significantly higher in the group of patients that received GnRH-a in the luteal phase (43.2%, 111/257) than in the group that were not administered this hormone agonist (34.4%, 95/276) (P = 0.04; OR = 1.44, 95% CI 1.01, 2.05). There was heterogeneity in this comparison (Breslow-Day = 8.94, df = 2, P = 0.01; Cochran Q = 8.74, df = 2, P = 0.01; I^2 ^= 77.1%, 95% CI = 0% to 90.9%)

### Ongoing pregnancy rate (Fig 4)

**Figure 4:**
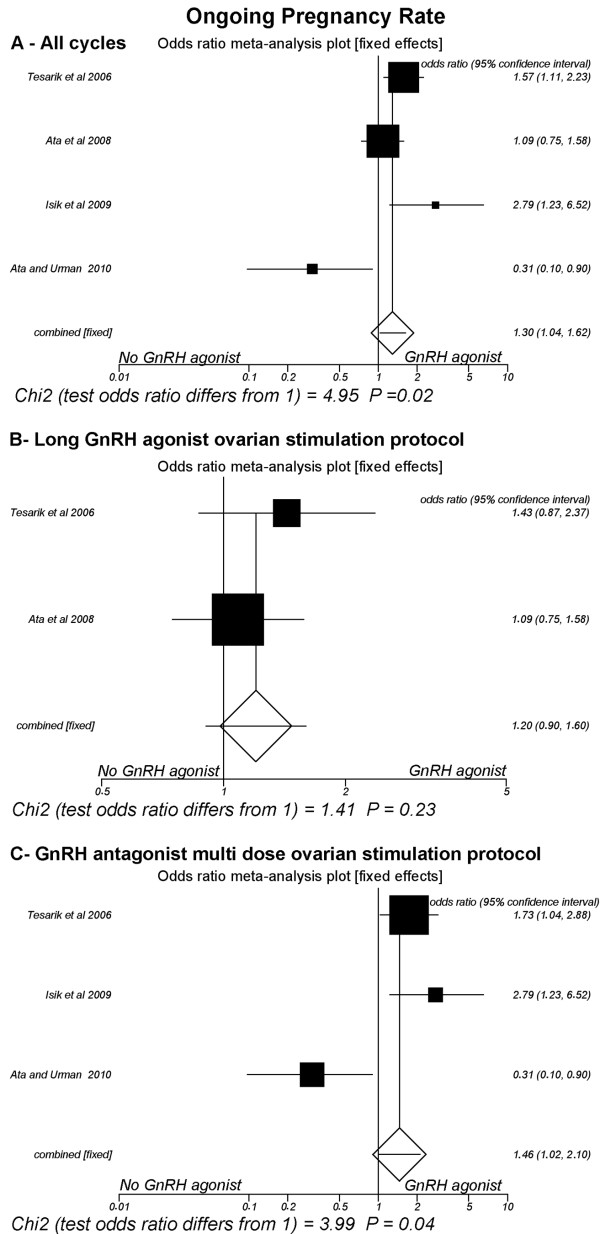
**Fixed-effect model**. Ongoing clinical pregnancy rates. A: All cycles; B: Only cycles with long GnRH agonist ovarian stimulation protocol; C: Only cycles with GnRH antagonist multi low-dose ovarian stimulation.

Four studies were included [15,18,23,24]. The pooled ongoing pregnancy rate was significantly greater in the group of patients that received GnRH-a in the luteal phase (37%, 253/683) than in the group that did not receive it (31.1%, 219/703) (P = 0.02; OR = 1.29, 95% CI 1.03, 1.62), a heterogeneous comparison (Breslow-Day = 14.94, df = 3, P = 0.001; Cochran Q = 14.23, df = 3, P = 0.002; I^2 ^= 78.9%, 95% CI = 7.9% to 90.2%).

However, in the subgroup of trials where the long GnRH-a ovarian stimulation protocol was used [18,24], the pooled ongoing pregnancy rate was not significantly different between the patients that received (36.4%, 155/426) versus the group that did not receive GnRH-a in the luteal phase (32.3%, 138/427) (P = 0.23; OR = 1.20, 95% CI 0.90, 1.59). There was no heterogeneity in this comparison (Breslow-Day = 0.84, df = 1, P = 0.35; Cochran Q = 0.84, df = 3, P = 0.25). On the other hand, in the subgroup of trials where the GnRH-ant ovarian stimulation protocol was used [15,18,23], the pooled ongoing pregnancy rate was significantly elevated in the group of patients that received GnRH-a in the luteal phase (38.1%, 98/257) in relation to the group that did not receive it (29.3%, 81/276) (P = 0.04; OR = 1.46, 95% CI 1.02, 2.10), a comparison with heterogeneity (Breslow-Day = 13.6, df = 2, P = 0.001; Cochran Q = 12.7, df = 2, P = 0.001; I^2 ^= 84.4%, 95% CI = 16.9% to 93.1%).

## Discussion

The increasing volume of information has stimulated a growing need for reviews of the medical literature. Meta-analysis differs from the narrative review by its rigorous and complete quantitative and qualitative methodological approaches. It is an analytical approach where different and independent studies are integrated and the results combined into a unique common result. When it is compared with narrative review, meta-analysis has the great advantage of being less influenced by the personal opinion of the reviewer thus providing impartial conclusions. Moreover, all of the results can easily be recalculated and compared with the authors' conclusions. The meta-analysis, even when not producing definite conclusions about the utility of a treatment, can support the necessity for new randomized trials on the subject. Different RCTs evaluating the effects of luteal GnRH-a administration on clinical outcomes have been published recently but with divergent conclusions [15,16,18,23,24]. Therefore, given the clinical potential of this practise, a review about this subject would seem to be helpful.

It has been suggested that a meta-analysis should be patient oriented, i.e. primary outcomes should be clinical results, which predict a better outcome and/or a more advantageous cost/effectiveness ratio. In this type of meta-analysis, GnRH-a administration in the luteal phase produced a favorable effect not only on the general implantation rate but also on the implantation rate for both subgroups. In addition, luteal GnRH-a treatment produced a significantly higher clinical pregnancy rate in the group administered the GnRH-ant multi-dose ovarian stimulation protocol. On the basis of these data, it may seem attractive to consider administration of a single dose of GnRH-a in the IVF/ICSI luteal phase to improve clinical outcomes, especially in GnRH antagonist cycles. Other RCTs not included in this meta-analysis [19,20] yielded the same results. Although these trials were performed in different conditions (GnRH agonist administration was continued until 14 days after oocyte retrieval; GnRH agonist administration on the day of ovum pickup, on the day of embryo transfer and three days thereafter, respectively), their outcomes are clearly consistent with those of this meta-analysis (i.e. increase in implantation rate) and support the hypothesis that GnRH-a administration in luteal phase may be useful.

Nevertheless, meta-analysis also presents problems. Heterogeneity and insufficient power (low sample size) hinder the ability to draw inferences about the meta-analysis, which failed to show any statistically significant difference in the clinical pregnancy rate and ongoing (in GnRH-a long protocol cycles) pregnancy rate. This observation can be related to a small cumulative sample size. In the long GnRH-a ovarian stimulation protocol, based on the CPR per transfer obtained in the groups with and without luteal GnRH-a (42%, 217/516 and 36.4%, 188/517), the ability to detect a difference of 5% with a power of 80% would require around 2450 patients to reach a definitive conclusion, i.e. a sample size above the total number included. Similarly, still In the long GnRH-a ovarian stimulation protocol, based on the ongoing pregnancy rates obtained in the groups with and without luteal GnRH-a (36.4%, 155/426 and 32.3%, 138/427), detecting a 5% difference with 80% power would require around 4300 patients to draw a definitive conclusion. Thus, for a more consistent conclusion, this meta-analysis guides researchers to wait for the results of new RCTs that have more information about clinical parameters.

One must be aware of the fact that a number of other significant predictors of the outcomes exist in an individual patient. Given that heterogeneity was observed in most of the comparisons carried out in this meta-analysis, a detailing of these studies should be made. Tesarik et al. [18] asserts that the effect of luteal-phase GnRH agonist administration should be interpreted in the context of luteal-phase support. However, it can be seen that the luteal phases were differently managed among the trials: Tesarik et al. [18]: Vaginal micronized progesterone(400 mg) + r-HCG (single dose) +E2 valerate; Ata et al [24] and Ata and Urman [23]: Vaginal progesterone gel/90 mg; Isik et al. [15]: Vaginal micronized progesterone(600 mg) + HCG(single dose); Razieh et al. [16]: Vaginal micronized progesterone(800 mg). This difference among the populations may have contributed to the divergent results obtained and, consequently, to the heterogeneity observed in this meta-analysis. Again, future controlled trials will clarify this issue.

The beneficial effects on clinical variables observed herein, despite the differences in the luteal-phase background, and the fact the drug can be easily prescribed, make the possibility of administering GnRH-a in ICSI luteal phase support appear more attractive. However, there is a need for discussion regarding the possible mechanisms of GnRH-a luteal action. The hypothesis that GnRH-a exerts a direct beneficial effect on the embryos [17] is supported by different observations. Animal experiments suggest that GnRH-a can improve the in vitro development of the embryo [26-29]. In addition, GnRH appears to exert regulatory activity in the synthesis and secretion of HCG by pre-implanted embryos and by the placenta [30,31]. Tesarik et al. [18] suggest possible direct effects on the embryo on account of observation of higher levels of serum ß-HCG in the overall group of patients who achieved a pregnancy after luteal-phase administration of GnRH-a as compared with placebo. On the other hand, given that the medication is administered in the presence of the embryos, such improvement in clinical outcomes should be carefully weighed against possible harmful effects on the health of resulting children. The relatively secure notion that exposure of embryos to GnRH-a is not prejudicial is based on a series of case reports on its accidental administration to pregnant women that, in general, report only on early postnatal examination of the children [10-14]. Thus, further long-term follow-ups of such children are still necessary to elucidate this point.

A direct action on uterine tissue may also be responsible for the effects of GnRH-a in the luteal phase. A presence of GnRH receptor with a dynamic pattern (more intense in the luteal phase) was demonstrated in human endometrium both in the epithelium and stroma [32-34]. Moreover, it has been also reported that GnRH and GnRH-a can alter the activity of matrix metalloproteinases, involved in tissue remodeling (matrix degradation) inherent to the process of endometrial trophoblast invasion [35,36], and induce apoptosis in endometrial cells in vitro [37,38]. However, in vitro GnRH analogues do not seem to have significant influence on the extent of decidualization of endometrial stromal cells in vitro [32] nor do they have major direct effects on gene expression of human endometrial epithelial cells [39]. Thus, despite the studies suggesting a direct action on endometrial function, clinically relevant endometrial effects of the GnRH analogues still need to be established.

The corpus luteum is another possible GnRH-a target in the luteal phase, though it is questioned whether such action would occur through the secretion of pituitary hormones or by direct action in the ovary. In cycles with GnRH-ant it is speculated that stimulation of corpus luteum activity by GnRH-a may result from stimulation of LH secretion, given that, despite the blockade, the pituitary remains responsive to the administration of GnRH or GnRH-a [15]. By contrary reasoning, in the long GnRH-a ovarian stimulation protocol, such action would likely be directly on the corpus luteus, since the pituitary blockade may still be present at the moment of GnRH-a administration [24]. In any case, independent of the probable action mechanism, Tesarik et al. [18] observed under both protocols, long GnRH-a ovarian stimulation or GnRH-ant multi low-dose ovarian stimulation, the increase in serum concentrations of E2 and progesterone in the luteal phase of patients that received GnRH-a 6 days after ICSI relative to those that were administered a placebo. Nevertheless, Hugues et al. [40], in GnRH-ant cycles, observed no differences in the hormonal profile of the luteal phase after GnRH-a administration.

In conclusion, the findings of this meta-analysis demonstrate that the luteal-phase single-dose GnRH-a administration can improve clinical outcomes after ICSI. However, by considering the heterogeneity between the trials, it seems premature to recommend the use of GnRH-a in the luteal phase. Additional randomized controlled trials are necessary before evidence-based recommendations can be provided. Protocols, safety, side effects and exact action mechanism(s) should de analyzed before the adoption of GnRH-a administration in the luteal phase.

## Competing interests

The authors declare that they have no competing interests.

## Authors' contributions

JBAO was responsible for designing and coordinating the study. All authors were responsible for data collection, data analysis, and data interpretation in the manuscript. JBAO, RB, MC and JF were responsible for the statistical work and for writing the manuscript. JF was responsible for reviewing the manuscript. All authors read and approved the final manuscript.
